# Novel Pectic Polysaccharides Isolated from Immature Honey Pomelo Fruit with High Immunomodulatory Activity

**DOI:** 10.3390/molecules27238573

**Published:** 2022-12-05

**Authors:** Tao Hou, Shenglan Guo, Zhuokun Liu, Hongyu Lin, Yu Song, Qiqi Li, Xin Mao, Wencan Wang, Yong Cao, Guo Liu

**Affiliations:** 1 Guangdong Provincial Key Laboratory of Nutraceuticals and Functional Foods, College of Food Sciences, South China Agricultural University, Guangzhou 510642, China; 2 Guangzhou Shuke Industrial Co., Ltd., Guangzhou 510642, China; 3 Chongqing Sweet Pet Products Co., Ltd., Chongqing 402160, China

**Keywords:** immature honey pomelo fruit, pectic polysaccharides, structural characterization, immunomodulatory activity and mechanism

## Abstract

A novel pectic polysaccharide (HPP-1) with high immunomodulatory activity was extracted and isolated from the immature honey pomelo fruit (*Citrus grandis*). Characterization of its chemical structure indicated that HPP-1 had a molecular weight of 59,024 D. In addition, HPP-1 was primarily composed of rhamnose, arabinose, fucose, mannose, and galactose at a molar ratio of 1.00:11.12:2.26:0.56:6.40. Fourier-transform infrared spectroscopy, periodic acid oxidation, and Smith degradation results showed that HPP-1 had α- and β-glycosidic linkages and 1 → 2, 1 → 4, 1 → 6, and 1 → 3 glycosidic bonds. ^13^C NMR and ^1^H NMR analyses revealed that the main glycogroups included 1,4-D-GalA, 1,6-β-D-Gal, 1,6-β-D-Man, 1,3-α-L-Ara, and 1,2-α-L-Rha. Immunomodulatory bioactivity analysis using a macrophage RAW264.7 model in vitro revealed that NO, TNF-α, and IL-6 secretions were all considerably increased by HPP-1. Moreover, RT-PCR results showed that HPP-1-induced iNOS, TNF-α, and IL-6 expression was significantly increased in macrophages. HPP-1-mediated activation in macrophages was due to the stimulation of the NF-κB and MAPK signaling pathways based on western blot analyses. HPP-1 extracted from immature honey pomelo fruit has potential applications as an immunomodulatory supplement.

## 1. Introduction

Immature honey pomelo (*Citrus grandis*) fruit (IPF) is a by-product of pomelo cultivation, which is in the middle of its growth cycle in April. A small proportion of IPF is used in traditional Chinese medicine, but the majority of IPF is discarded, which contributes to environmental pollution [1]. However, our previous research suggests that IPF is rich in bioactive substances such as essential oils, naringin, and pectin, which have considerable medicinal and commercial value. Essential oils and naringin have good antioxidant activity [2], yet research on the bioactive substances of IPF remains limited. Pectin, a polysaccharide straight-chain compound, is an abundant and multifunctional component of terrestrial plant cell walls. It has a high functional value (gelling, thickening, emulsification, and stabilization) and is widely used as a food and drug gelling agent [3,4]. Citrus pectin has strong biological activities, such as immunomodulatory [5,6], antioxidant [7], anti-inflammatory [8], anti-cancer [9], heavy metal adsorption [10], drug transport [11], and other properties [12]. Despite numerous studies on pectin, information on its structure and immune activity in IPF is limited.

Polysaccharides have been widely reported to stimulate macrophages, enhance their phagocytic function, and release various cytokines such as interleukins (IL-1β, IL-6, IL-8) and tumor necrosis factor (TNF-α), as well as nitric oxide and reactive oxygen species (ROS), thereby supporting the body’s immunity [13,14,15,16]. Some plant polysaccharides increase the secretion of nitric oxide (NO) and the synthesis of cytokines, which in turn increase macrophage action against pathogenic microorganisms and tumorigenesis [6]. NO is considered to be the primary effector molecule produced by macrophages. When macrophages are stimulated and activated, they secrete a series of chemokines and cytokines. This plays an important role in activating the immune response and immunomodulation of the body [17]. TNF-α is a cytokine capable of killing tumors to cause hemorrhagic necrosis and has clear antitumor activity. Moreover, TNF-α is produced mainly from activated macrophages and is a very important cytokine in the antitumor immune response, and is a common and important indicator of macrophage activity [18]. IL-6 is a type of pleiotropic cytokine and it is involved in the body’s immune defense, as well as in promoting the growth and differentiation of primary bone marrow-derived cells [19]. Polysaccharides may bind to specific membrane receptors in macrophages and activate immune response transduction pathways via various signals. Based on previous studies, activator protein 1 (AP-1) and MAPK activity in the macrophage RAW264.7 were stimulated by polysaccharides from *Platycodon grandiflorus* (PG). Moreover, further research revealed that PG activates three subsets of MAPKs (ERK1/2, SAPK/JNK, and p38 MAPK) through increasing the DNA-binding activity of AP-1 [20,21]. However, the exact molecular mechanism through which IPF pectin activates macrophages remains unclear.

In this study, a crude IPF pectin was obtained from the IPF residue after the extraction of essential oil and naringin. Honey pomelo pectic polysaccharides (HPP-1) were systematically purified using anion exchange and gel permeation chromatography. The structure characterization of HPP-1 was investigated systematically by UV spectroscopy, chromatography, mass spectrometry, nuclear magnetic resonance (NMR), and other analytical techniques, whereas the immune activity and pattern recognition receptors of HPP-1 was evaluated using a mouse macrophage RAW 264.7 immune model in vitro. We analyzed the effect of HPP-1 on the pinocytic and phagocytic capacity, production of TNF-a, NO, and IL-6, and relevant mRNA expression. Furthermore, western blots were used to reveal the mechanism of HPP-1 immunoreactivity. In addition, the possible signaling pathways involved in HPP-1 activating macrophages were discussed, and these should all help further clarify the structure-effective relationship of HPP-1. The results provide valuable information for the application of IPF extracts in immunomodulatory activity.

## 2. Results and Discussion

### 2.1. Extraction and Purification of Pectic Polysaccharides from IPF

Crude IPF pectin was obtained from the residue after essential separation with a yield of 20.23% ± 0.66% (*w*/*w*). Crude pectin was composed of 71.17% total sugar (59.90% galacturonic acid), 1.93% protein, and 4.33% ash. The degree of esterification was 44.65%, indicating that the compound was a low-ester pectin. Based on previous reports, pectin with esterification below 10% has anti-cancer properties, whereas high-ester pectin has anti-inflammatory properties [8].

**Figure 1:**
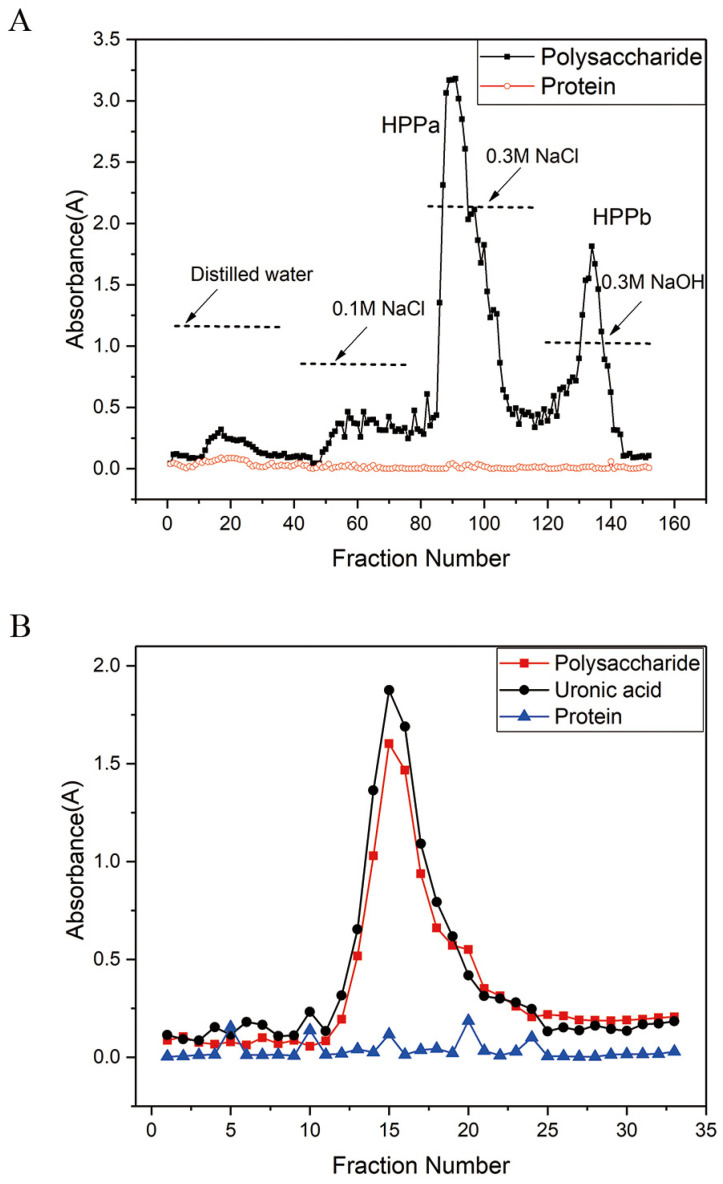
Chromatography of HPP-1 from honey pomelo pectin (HPP) by (**A**) DEAE-52 cellulose and (**B**) Superdex-G200.

When the extracted pectin was purified, two independent peaks were revealed: HPP-a (eluted by 0.3 M NaCl) and HPP-b (eluted by 0.3 M NaOH, Figure 1A). When HPP-a was further purified, a single peak of HPP-1 was observed, which contained a galacturonic acid reaction, but without protein (Figure 1B). Furthermore, a >98%-pure HPP-1 fraction was obtained.

### 2.2. Analysis of the HPP-1 Structure

#### 2.2.1. UV Scan and Molecular Mass Detection

**Figure 2:**
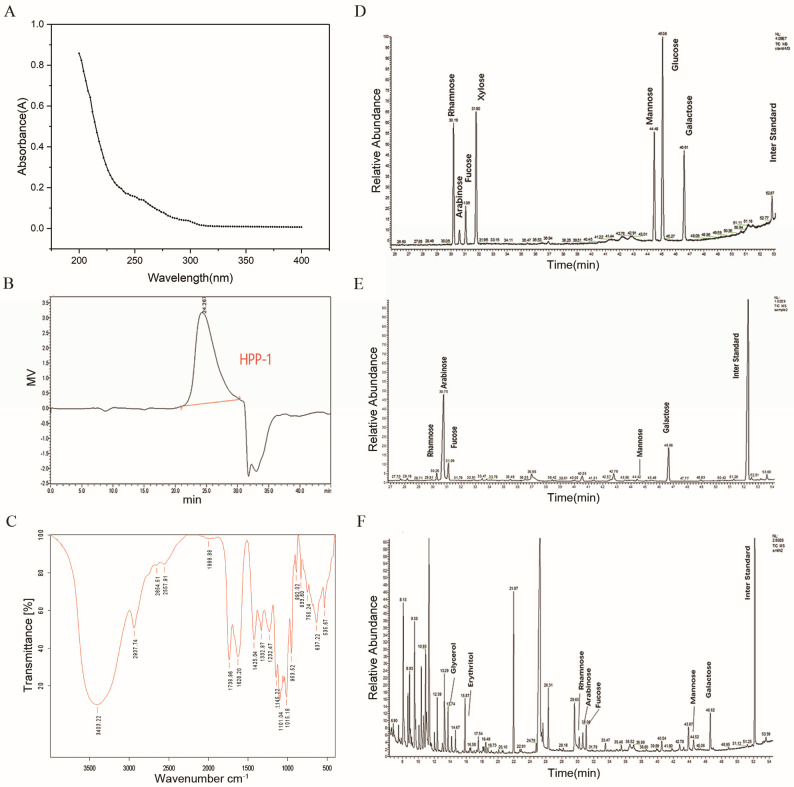

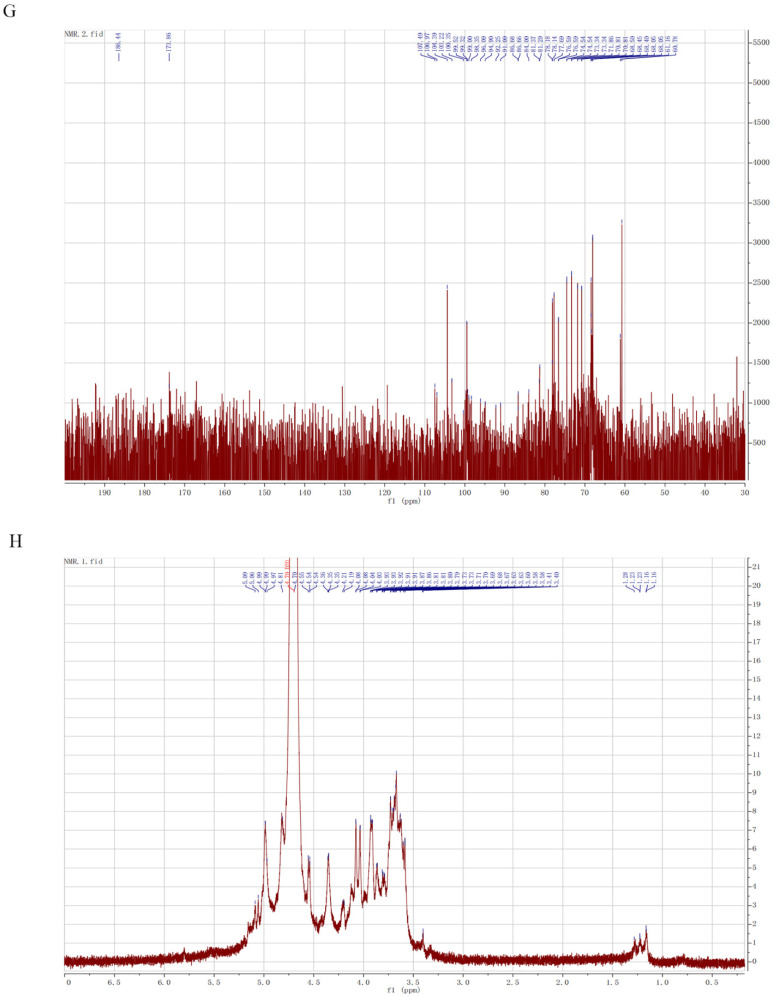
Chromatography results of HPP-1 obtained from (**A**) UV spectrum, (**B**) HPGPC, (**C**) FT-IR spectrum, gas chromatography-mass spectrometry (GC-MS) of (**D**) standard monosaccharide, and (**E**) monosaccharide composition of HPP-1. (**F**) GC of HPP-1 after Smith degradation. HPP-1 (**G**) ^13^C NMR spectrum and (**H**) ^1^H NMR spectrum.

The UV spectral scan of HPP-1 revealed no evident characteristic absorption peaks of amino acids or proteins (i.e., near 260 and 280 nm; Figure 2A). However, these spectra were consistent with the characteristic absorption spectral peaks of polysaccharides [22]. These results indicate that the separated HPP-1 fraction did not contain protein, suggesting that the protein in crude HPP could be completely removed during purification.

The structure of HPP-1 was further analyzed by GPC. The GPC results showed that HPP-1 had a single symmetric peak with a molecular mass of 59,024 Da (Figure 2B), indicating that HPP-1 has a small molecular mass. In general, the average molecular mass of pectin is between 50,000 and 150,000 Da [23]. It was hypothesized that pectin is a concentrated pectin fragment molecule, which requires further experimental confirmation.

#### 2.2.2. FT-IR Spectrum

The FTIR spectrum of HPP-1 is shown in Figure 2C. Absorption peaks were observed at 3403.22 cm^−1^, and the weak peaks at 2937.74 and 145.22 cm^−1^ correspond to characteristic absorption peaks of polysaccharides, which are associated with O-H, C-H, and C-O-C glycosidic stretching vibrations [24]. These results indicated that HPP-1 is a polysaccharide. Furthermore, absorption peaks at 1738.96 and 1628.20 cm^−1^ indicated the C=O stretching vibration of methylated carboxyl groups and free carboxyl groups on the HPP-1 sugar chain, respectively, and the absorption peak at 1015.18 cm^−1^ corresponded to pyranose structures [25]. The absorption peak at 1738.96 cm^−1^ likely represents the stretching vibration of uronic acid, whereas the peaks at 892.02 and 833.60 cm^−1^ represent β-D-mannitose and glycoside bonds in the alpha configuration, respectively [25]. Therefore, HPP-1 contains α-configurational and β-configurational glycosidic bonds.

#### 2.2.3. Monosaccharide Composition Assay

The GC–MS results for the monosaccharide standard and HPP-1 are shown in Figure 2D and Figure 2E, respectively.

By comparing the elution time of HPP-1 with that of monosaccharide standard, HPP-1 was composed of rhamnose (Rha), arabinose (Ara), fucose (Fuc), mannose (Man), and galactose (Gal), with a molar ratio of 1.00:11.12:2.26:0.56:6.40. The presence of mannose and fucose indicates the presence of an RG II region in HPP-1. Arabinose and galactose were the main components of HPP-1, and the arabinose and galactose contents were higher than those of rhamnose. This finding indicated that the RG I regional fragment existed simultaneously, and arabinose, galactose, and Arabian galactose were the main components of the RG I fragment branch chain [26].

#### 2.2.4. Periodate Oxidation-Smith Degradation Analysis

Periodate oxidation revealed that 0.1150 mmol of periodate was consumed by HPP-1 and 0.0306 mmol of methane acid was produced, indicating the presence of (1 → 6)-linked glucoside residues (Figure 2F). The consumption of sodium periodate was higher than that of formic acid, indicating additional (1 → 2) and (1 → 4) glycoside bond types [27].

Glycerol, erythritol, rhamnose, arabinose, fucose, mannose, and galactose were observed after HPP-1 underwent Smith degradation (Figure 2F). The formation of glycerol indicates (1 → 2)-or (1 → 6)-linked glycosidic bonds, whereas erythritol indicates (1 → 4)-linked glycosidic bonds. In addition, the formation of rhamnose, arabinose, galactose, mannose, and galactose indicates the presence of (1 → 3)-linked glycosidic bonds [18].

#### 2.2.5. NMR Analysis

The ^13^C NMR and ^1^H NMR spectra of HPP-1 are shown in Figure 2G and Figure 2H, respectively. The resonance signals between *δ* 95.0 and 110.0 in ^13^C NMR belong to the anomeric carbon atoms of monosaccharides [24,28]. Five anomeric carbon atoms (99.51, 103.22, 104.39, 107.47, and 173.21 ppm) were detected in HPP-1. In ^1^H NMR, the protons between *δ* 3.5 and 5.5 indicates anomeric hydrogen [18]. The chemical shift of α-configurational and β-configurational sugars was greater than 4.90 and less than 4.90 ppm, respectively, indicating the presence of α and β-configurations. This finding is consistent with the FTIR results. A small peak at *δ* 173.21 ppm indicated uronic acid, which was the main chain of the pectin structure, indicating the existence of 1,4-D-GalA [29]. The signals at *δ* 68.742 and *δ* 67.98 ppm, combined with infrared spectrum and monosaccharide composition, indicate the presence of 1,4-β-D-Gal and 1,6-β-D-Man [18]. The peaks at *δ* 107.47 and *δ* 82.36 ppm are characteristic of furanose (arabinose). Peaks at 16.73 and 16.49 ppm indicate hydrogen on rhamnose C6. Combined with previous reports [18,24,27,30], monosaccharide composition, and Smith degradation results, we identified 1,3-α-L-Ara and 1,2-α-L-Rha glycosylates in HPP-1.

1,4-D-GalA, 1,4-D-GalA-(1,2-α-Rha), 1,2-α-Rha, 1,2,4-α-Rha, 1,4-β-D-Gal, t-β-D-Gal, and α-L-Ara sugar residues were detected in pumpkin acid polysaccharides, which had a pectic polysaccharide RG I structure similar to that of HPP-1 [29]. The structure of pectic polysaccharides has a structure–activity relationship with their immunomodulatory activity [6].

### 2.3. Immunomodulatory Activities of HPP-1 on RAW264.7 Cells

#### 2.3.1. Effect of HPP-1 on RAW264.7 Cell Viability

**Figure 3:**
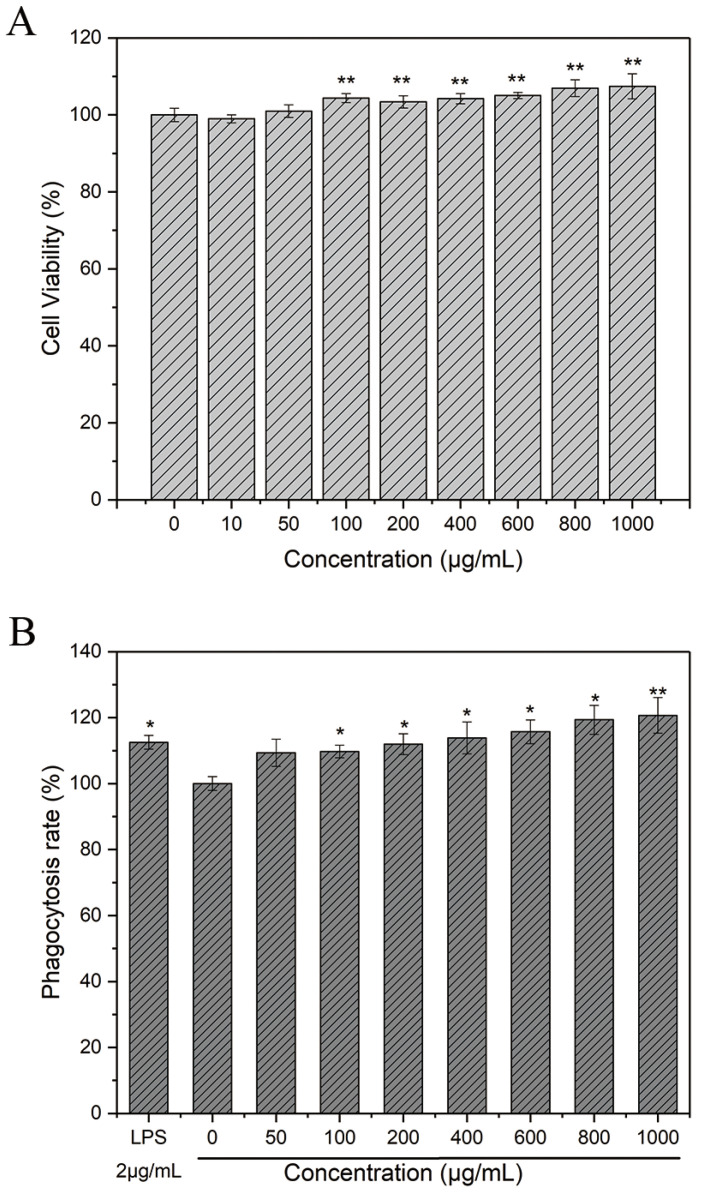
(**A**) Effect of HPP-1 on the viability of RAW 264.7 cells. (**B**) Effect of HPP-1 on the uptake of neutral red by RAW 264.7 cells. Significant differences with control cells were designated as * *p* < 0.05 or ** *p* < 0.01.

After being treated with HPP-1 (10, 50, 100, 200, 400, 600, 800, and 1000 μg/mL) for 24 h [31], the results indicated that RAW 264.7 cells showed no toxicity at concentrations below 1000 μg/mL of HPP-1 (Figure 3A). At concentrations of 600–1000 μg/mL, HPP-1 significantly improved cell viability and promoted macrophage proliferation.

#### 2.3.2. Effect of HPP-1 on the Phagocytic Capacities of RAW264.7 Cells

Neutral red is an effective acid–base indicator of living cells that can react with lysosomes to produce red substances [25]. The amount of neutral red entering the cell varies depending on the state of the living cell and can be used to assess the ability of macrophages to produce pinocytes.

After treatment with HPP-1, the intensity of neutral red cell absorption increased compared with that in the control group (Figure 3B). The phagocytic rate of cells at 200–1000 μg/mL was significantly higher than that of the control group (*p <* 0.05). This result indicated that HPP-1 enhanced the phagocytic capacity of neutral red in murine macrophages. This finding is in accordance with previous findings, that is, polysaccharides from purple sweet potato and *Chrysanthemum indicum* stem polysaccharides could promote phagocytosis of neutral red by macrophages [32,33]. Acidic polysaccharides derived from *Cucurbita moschata Duch*, *Persimmon Leaves*, and *Helicteres Angustifolia* L. are pectic polysaccharides containing D-galacturonic acid, as previously reported. Furthermore, the improvements in macrophage phagocytic capacity were identified [34,35,36].

#### 2.3.3. Effects of HPP-1 on Macrophage NO, TNF-α, and IL-6 Production

**Figure 4:**
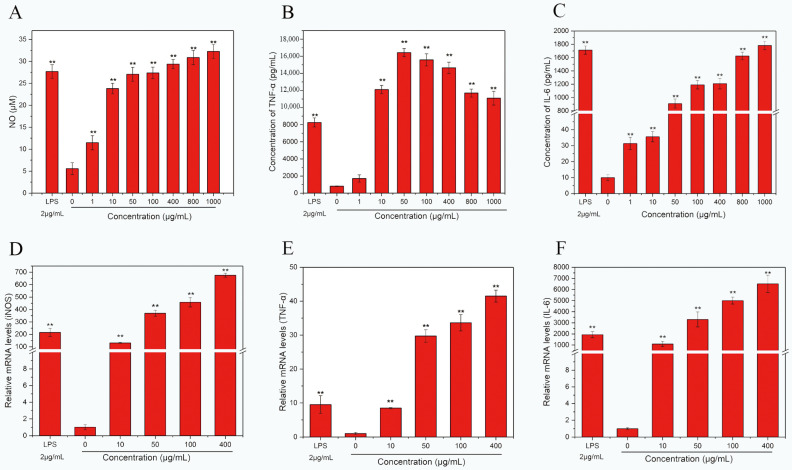
Effects of HPP-1 on cytokine secretion of (**A**) NO, (**B**) TNF-α, and (**C**) IL-6, mRNA levels of (**D**) iNOS, (**E**) TNF-α, and (**F**) IL-6. The group without HPP-1 was used as the negative control, and LPS (2 μg/mL) was used as the positive control. Significant differences with control cells were designated as ** *p* < 0.01.

HPP-1 fractions stimulated NO and IL-6 secretion from macrophages at concentrations ranging from 1 to 1000 μg/mL in a dose-dependent manner (Figure 4A–C). At increasing concentrations of HPP-1, TNF-α secretion showed an upward and then a downward trend. TNF-α is not only an important cytokine in immune regulation but is also a key cytokine involved in inflammation [37], cellular homeostasis, tumor progression, and insulin resistance in individuals with obesity and diabetes [38,39]. At 50 μg/mL of HPP-1, the NO secretion level reached 27.06 ± 1.60 μM, which was significantly higher than that of the blank control (*p*< 0.01) and reached the NO secretion effect of 27.68 ± 1.55 μM of the LPS-positive treatment group (2 μg/mL, Figure 4A). As HPP-1 concentrations increased, NO secretion significantly increased. When the concentration of HPP-1 reached 400 μg/mL, NO secretion in the experimental group was higher than that in the LPS-positive control group (*p* < 0.01). This finding is in accordance with those of previous studies [27,36].

Compared with the control group, increasing concentrations of HPP-1 from 1 to 1000 μg/mL significantly promoted TNF-α secretion by macrophages (Figure 4B). TNF-α secretion exceeded that of the positive and blank control groups at HPP-1 concentrations of 100–1000 μg/mL (*p* < 0.01). The results showed that 50 μg/mL HPP-1 stimulated the greatest secretion of TNF-α in macrophages, which was substantially higher than the amount stimulated by the same concentration of the purified fraction of fungal monkey head mushroom polysaccharide [24]. When the concentration was greater than 50 μg/mL, TNF-α secretion slowed but remained higher than that of the LPS-stimulated blank control group.

HPP-1 also increased macrophage IL-6 secretion at concentrations ranging from 1 to 1000 μg/mL (Figure 4C). At concentrations of 1–10 μg/mL, the secretion of IL-6 was 30–40 times that of the blank control (*p* < 0.01). IL-6 secretion reached 1782.17 ± 63.12 pg/mL at HPP-1 concentrations of 1000 μg/mL, exceeding that of the positive control group (1713.09 ± 62.90 pg/mL).

#### 2.3.4. Effects of HPP-1 on iNOS, TNF-α, and IL-6 mRNA Levels in Macrophages

Expression of immune-related cytokines is associated with activation and immunomodulatory effects of macrophages [40].

HPP-1 increased the expression levels of iNOS, TNF-α, and IL-6 in macrophages at concentrations of 10–400 μg/mL in a dose-dependent manner (Figure 4D–F). At 50 μg/mL, the expression of iNOS, TNF-α, and IL-6 was 370, 29 and 3300 times higher than that of the control group, respectively (*p* < 0.01), indicating that the HPP-1 fraction was more effective in upregulating the expression levels of iNOS, TNF-α, and IL-6. When the stimulation concentration reached 400 μg/mL, the expression levels of iNOS, TNF-α, and IL-6 were only 3–4 times higher than those in the LPS-positive group. High mRNA expression can induce the secretion of corresponding immune factors, thereby improving the immune capacity [41]. In addition, *TNF-α* expression was not positively correlated with TNF-α secretion concentration, probably because transcription is only the first step of TNF-α formation and is affected by post-transcriptional regulation [42,43]. Pectic polysaccharides from other plants have the same effects on macrophages. Gavlighi [44] used enzymatic extraction and purification of pectin polysaccharides from pomegranate peel. The fraction with the best immune effect significantly upregulated the expression levels of *iNOS*, *IL-1β*, *TNF-α*, *IL-6*, and *IL-10* in a concentration-dependent manner. Moreover, western blotting showed that the immune effect fraction could affect the expression of cytokine levels and exert immunomodulatory effects related to the NF-κB and MAPK signaling pathways. The immunomodulatory molecular mechanism of HPP-1 must be further elucidated by studying signaling pathways.

### 2.4. Effect of HPP-1 on Nf-κB and MAPKs Signaling Pathways in Murine Macrophages

**Figure 5:**
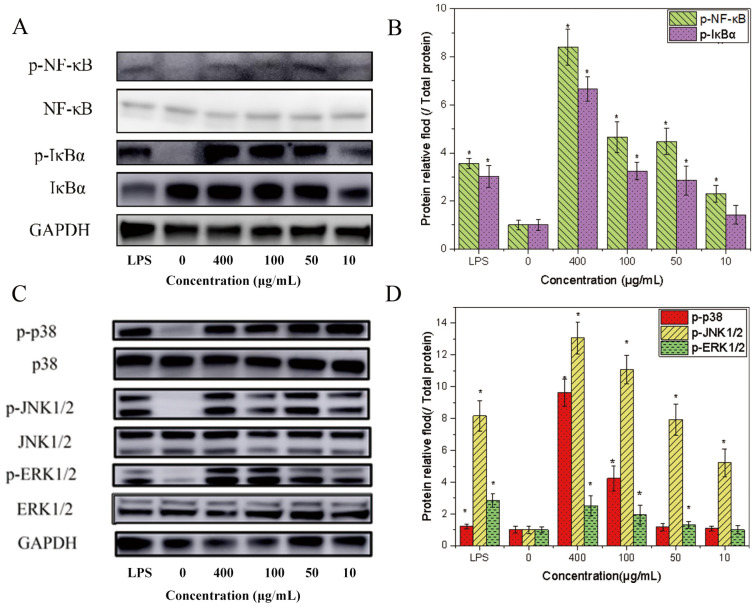
Effects of HPP-1 treatment on Nf-κB and MAPK signaling pathways. (**A**) Representative Western blotting bands and (**B**) quantitative analysis of NF-κB/IκBα protein levels (values were normalized to the GAPDH level). (**C**) Representative Western blotting bands and (**D**) quantitative analysis of p-p38/p38, p-pJNK/pJNK, and p-AKT/AKT protein levels. The group without HPP-1 was used as the negative control, and LPS (2 μg/mL) was used as the positive control. Significant differences with control cells were designated as * *p* < 0.05.

Based on previous literature, several intracellular signal transduction routes have been shown to activate macrophages. NF-κB is an important gene expression regulator [45]. Macrophages usually remain in the cytoplasm because of their non-covalent binding to the NF-κB-IκB trimer. Upon activation of NF-κB signaling, IκBα serine residues are phosphorylated, which causes NF-κB to separate from IκBα and move to the nucleus as an activated transcription factor [46]. The binding sites of the p50–p65 dimer were revealed after IκB dissociation, allowing them to connect to the κB motif. The NF-κB p65 subunit then moves from the cytoplasm to the nucleus, inducing the transcription of a range of genes, including iNOS, ROS, and macrophage-related cytokines [47]. We used these samples to activate RAW 264.7 macrophage cells via western blot analysis to determine whether the NF-κB (NF-κB/IκBα) signaling pathway was implicated in HPP-1 (10–400 μg/mL) or LPS (2 μg/mL). NF-κB and IκBα protein levels increased in a dose-dependent manner with an increase in HPP-1 (*p* < 0.01), exhibiting significantly higher expression levels than those in the negative control group (Figure 5A,B; lane 2). Moreover, when the dose of HPP-1 reached 50 μg/mL, both protein expression levels were higher than those in the positive control group (Figure 5A,B; lane 1).

MAPKs are a group of serine/threonine-specific protein kinases that play a role in the activation of NF-κB and in cellular responses to diverse extracellular stimuli [48]. The MAPK family is divided into three subgroups: ERK1/2, p38, and JNK1/2 [48]. NF-κB is activated by several intracellular signaling pathways, including the MAPK pathway [48]. We evaluated the effect of HPP-1 on the MAPKS signaling pathway by western blotting. The addition of HPP-1 (100–400 μg/mL, 50–400 μg/mL, and 10–400 μg/mL) significantly increased the phosphorylation levels of p38, ERK1/2, and JNK1/2 compared to those in the control group (*p* < 0.01, Figure 5C,D). However, for p38 and ERK1/2, no difference in phosphorylation levels was observed at low doses (10–50 μg/mL) of HPP-1, indicating that HPP-1 could stimulate the phosphorylation of JNK1/2 at relatively low concentrations. Furthermore, 100 μg/mL of HPP-1 increased the phosphorylation levels of p38 and JNK1/2 compared to the LPS control group, which showed stronger immune activation than related polysaccharides [14,44].

Collectively, these results indicate that HPP-1-mediated activation of macrophages is achieved by activation of the NF-κB and MAPK signaling pathways.

### 2.5. Pattern Recognition Receptors and Potential Molecular Mechanisms of HPP-1-Induced Macrophage Immunomodulation Activity

**Figure 6:**
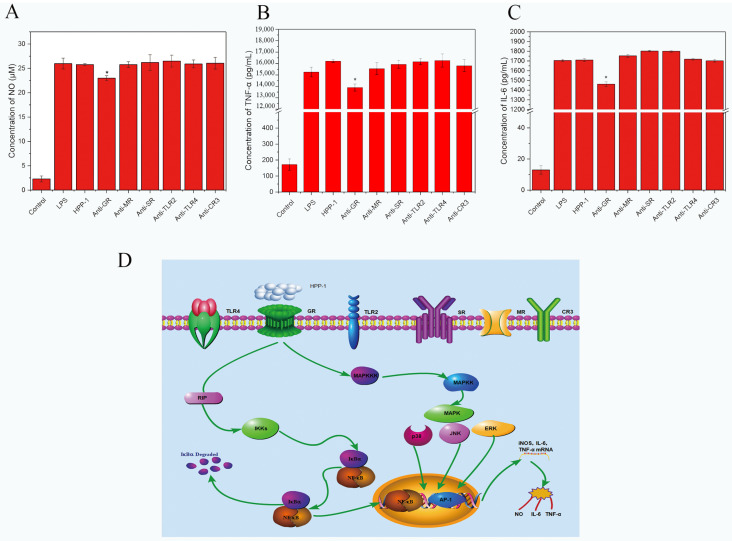
Effects of GR, MR, SR, TLR2, TLR4, and CR3 on the secretion of NO (**A**), TNF-α (**B**), and IL-6 (**C**) in RAW 264.7 cells. The group without HPP-1 was used as the negative control, and LPS (2 μg/mL) was used as the positive control. Potential signal transduction pathways involved in macrophage activation by HPP-1 (**D**). Significant differences with control cells were designated as * *p* < 0.05.

Plant polysaccharides interact with a variety of pattern recognition receptors on the surfaces of immune cells [38]. Pattern recognition receptors on macrophages can identify polysaccharides that activate macrophages to participate in immune regulation [49]. These receptors include complement receptor type 3 (CR3), Toll-like receptor (TLRs), β-glucan receptor (GR), mannose receptor (MR), and scavenger receptor (SR). When macrophage membrane receptors attach to glycosyl ligands on polysaccharides, a series of signaling cascades are activated [49]. NF-κB, PI3K/Akt, MAPKs, and MyD88/IRAK-1/TRAF-6 appear to be important signaling pathways in the regulation of macrophage cell immunity [50]. Thus, we used these antibodies to evaluate whether the immunomodulatory effects involved the participation of TLR2, CR3, TLR4, MR, GR, and SR of HPP-1. Compared to the group treated only with HPP-1, NO, IL-6, and TNF-α, levels were significantly decreased following anti-GR therapy (*p* < 0.01, Figure 6A–C). However, no reduction in NO, TNF-α, and IL-6 levels was observed in the groups treated with anti-CR3, anti-TLR4, anti-TLR2, anti-MR, or anti-SR. This result indicates that macrophage GR is a receptor of HPP-1, causing transcriptional factors to be activated and cytokines to be expressed in the presence of HPP-1. In addition, HPP-1 may contain structural fragments similar to β-glucan and initiate the same effects as β-glucan, which is widely used as a natural bioactive modulator in tumor immunotherapy [51]. However, this finding warrants further investigation.

The β-glucan receptor (GR) consists of the scavenger receptor (SR) [51], complement receptor type 3 (CR3) [52], lactosylceramide (Lac Cer) [53], and dendritic cell–associated C-type lectin-1 (Dectin-1) [54]. Moreover, Dectin-1 is a major β-glucan receptor in macrophages [55]. Dectin-1 pathways can activate numerous effects of polysaccharides on immunomodulation, anti-tumor processes, and anti-radiation functions [56].

Under stimulation by HPP-1, RAW 264.7, cells were likely turned on by MAPK/NF-κB signaling pathways via GR to activate transcription factors such as mRNA encoding iNOS, TNF-α, and IL-6, which are responsible for increasing NO, TNF-α, and IL-6 secretion (Figure 6D).

## 3. Methods and Materials

### 3.1. Materials and Chemicals

Dried IPF was obtained from Li Pomelo Guangdong Agricultural Science and Technology Co., Ltd. (Meizhou, Guangdong, China) and was stored at room temperature.

The murine macrophage cell line (RAW 264.7) was purchased from Kunming Cell Bank of the Chinese Academy of Sciences Culture Collection. DEAE-52 cellulose and Sephadex G-200 were obtained from GE Healthcare Life Science (Piscataway, NJ, USA). Monosaccharide standards (glucuronic acid, rhamnose, arabinose, fucose, xylose, mannose, glucose, galactose, and inositol) and LPS were obtained from Sigma-Aldrich Company (St. Louis, MO, USA). Dulbecco’s modified eagle’s medium (DMEM), fetal bovine serum (FBS), phosphate buffer saline (PBS, pH 7.4), penicillin, and streptomycin were purchased from Gibco Life Technologies (Grand Island, NY, USA). Antibodies (anti-mannose receptor (anti-MR), anti-scavenger receptor I (anti-SR), anti-toll-like receptor 2 (anti-TLR2), anti-toll-like receptor 4 (anti-TLR4), anti-beta glucan receptor (anti-GR), anti-complement receptor 3 (anti-CR3), nuclear factor-κ-gene binding (NF-κB), phospho-NF-κB (p-NF-κB), inhibitor of NF-κB (IκBα), phospho-IκBα (p-IκBα), extracellular regulated protein kinase (ERK1/2), phospho-ERK1/2 (p-ERK1/2), c-Jun N-terminal kinase (JNK1/2), phospho-JNK (p-JNK1/2), p38, phospho-p38 (p-p38), glyceraldehyde-3-phosphate dehydrogenase (GAPDH)) were obtained from Abcam, Inc. (Cambridge, MA, USA). Chemiluminescence (ECL) kit and NO-detecting kit were purchased from Beyotime Biotechnology Co., (Shanghai, China). Mouse IL-6 enzyme-linked immunosorbent assay (ELISA) kit and mouse TNF-α ELISA kit were obtained from Neobioscience Technology Co., Ltd. (Shenzhen, China). Standards of dextrans, uronic acid, phycite, glycerol, glycol reference, neutral red, 3-(4,5-dimethylthiazol-2-yl)-2,5-diphenyltetrazolium bromide (MTT) and Trizol were purchased from Macklin Biochemical Co., Ltd. (Shanghai, China). RNA Easy Fast Cell Kit, First Stand cDNA Synthesis Kit and FastStart Universal SYBR Green Master (ROX) were acquired from TIANGEN Biotech Co., Ltd. (Beijing, China).

### 3.2. Extraction of HPP

Approximately 1 kg of dried IPF fruit was ground into a 40 mesh powder and pretreated to extract essential oils and naringin using a novel continuous phase-transition extraction device [57], based on our previously reported methodology [2]. The resulting IPF residue was subjected to acid extraction and alcohol precipitation to extract pectin [58]. In brief, deionized water was adjusted to pH 1.5 using 0.5 M HCl. The IPF residue and 0.3–0.4% sodium hexametaphosphate were added to deionized water (pH 1.5) at a 1:20 solid-to-liquid ratio. This mixture was extracted at 90 °C for 90 min and centrifuged (9000 rpm) at 4 °C for 20 min. The resulting filtrate was concentrated to half its original volume at 55 °C under reduced pressure. This solution was then passed through an electrodialysis device (HMTECH-1220, Hangzhou, China) to remove salt.

The demineralized solution was mixed two times the volume of 95% ethanol and kept at 4 °C for 4 h, after which the alcohol precipitate was collected and washed three times with 95% ethanol. Finally, the precipitate was freeze-dried to obtain crude pectin (HPP).

### 3.3. Purification of Crude Pectin

HPP was purified using anion DEAE-52 exchange column chromatography based on previous methods [21], with the following modifications. HPP (500 mg) was loaded onto a DEAE-52 cellulose ion exchange column (2.6 × 50 cm) after being dissolved in 40 mL of deionized water. The DEAE-52 column was eluted with deionized water, 0.1 M NaCl, 0.3 M NaCl, and 0.3 M NaOH solution. The flow rate was set at 1 mL/min. The resulting eluate (5 mL/tube) was collected, and the total sugar and galacturonic acid contents were tested. Samples containing sugar and galacturonic acid were collected in a bag for dialysis (10 kDa) and dialyzed for 48 h at 4 °C in deionized water. Sugar and galacturonic acid contents were determined using previously described methods [59]. Two subfractions of HPP-1 (HPP-a and HPP-b) were obtained and freeze-dried. HPP-a, the most abundant component, was further studied.

In 5 mL of deionized water, 50 mg of HPP-a was dissolved before being placed on a Sephadex G-200 column (2.6 × 60 cm) at a 0.5 mL/min flow of deionized water.

### 3.4. Analysis of the HPP-1 Chemical Structure

#### 3.4.1. Ultraviolet Full Wavelength Scan

HPP-1 was dissolved in deionized water to a 0.1 mg/mL sample solution, and the protein content was measured using a UV3010 ultraviolet–visible spectrophotometer with continuous scanning at 200–400 nm based on previous methods [60].

#### 3.4.2. HPGPC Molecular Mass Detection

The molecular mass of HPP-1 was quantified using high-performance gel permeation chromatography (HPGPC) as previously described [28]. HPP-1 and dextran standards 5–670 kDa were mixed with deionized water to obtain a 2.0 mg/mL solution. A chromatographic column (TSK G-5000 connection G-3000 PWXL gel column) with a mobile phase of 0.02 M KH_2_PO_4_ solution was used. The column temperature and flow rate were set to 35 °C and 0.6 mL/min, respectively. The injection volume was 10 µL.

#### 3.4.3. Fourier Transform Infrared Spectroscopy (FT-IR) Analysis

The FT-IR spectrum of HPP-1 was obtained using a Nexus Fourier transform infrared spectrometer (Nicolet Nexus, Thermo Nicolet Company, Wilmington, DE, USA). Dry HPP-1 (1.0 mg) was mixed with 100 mg dried potassium bromide powder using an agate mortar. After grinding to a uniform consistency, the sample was pressed into thin slices using a tablet press and scanned from 4000 to 500 cm^−1^.

#### 3.4.4. Monosaccharide Composition

Pectic polysaccharides are composed of monosaccharides as structural units that are connected by glycosidic bonds. The glycosidic linkages were disrupted, and the derivatives were identified using high-temperature acid hydrolysis. The types of monosaccharide residues, their relative content, and their connection modes in pectic polysaccharides have been analyzed [30]. The composition of neutral sugars in HPP-1 was determined using gas chromatography–mass spectrometry [61]. In brief, 5.0 mg of the HPP-1 powder was added to 5.0 mL of 4 M trifluoroacetic acid and hydrolyzed in a sealed enclosure at 110 °C for 3 h. After hydrolysis, methanol (3.0 mL) was added, and the solution was concentrated under reduced pressure for 4–5 iterations. The HPP-1-concentrated solution was mixed with 10 mg of hydroxylamine hydrochloride and 1.0 mL of pyridine, and the resulting solution was heated at 90 °C for 30 min. Acetic anhydride (0.5 mL) was added to the mixture and the reaction was continued for an additional 30 min at 90 °C. The sample was then run through a chromatographic column (Agilent-technologies DB-5MS; 0.2 mm × 30 m × 0 25 μm, 110–160 °C at 2 °C/min, 160–220 °C at 1.5 °C/min, and then 220–260 °C at 5 °C/min, and kept at 250 °C for 5 min) equipped with a mass detector (MS) with a flow rate of 1.0 m L/min and an injection volume of 1 μL.

#### 3.4.5. Periodic Acid Oxidation and Smith Degradation Analysis

The position of the glycosidic linkages in HPP-1 was investigated using the periodate oxidation-Smith degradation method. Periodate can selectively oxidize and break the 1,2-diol group of sugar to produce the corresponding polyformic acid. Different monosaccharide connections produce different products [62]. Smith degradation is a process in which the oxidation products of periodate are reverted to stable polyhydroxyl compounds, hydrolyzed, and derived, and the hydrolyzed products were identified.

The type and content of glycosidic bond were determined from HPP-1 using previously published methods [63]. In brief, 10.0 mg HPP-1 was dissolved in 10 mL of 30 mM sodium periodate solution. After the sample had fully reacted for 10 h, 2 mL of the resulting oxidation solution and 0.1 mL of ethylene glycol were mixed for 10 min. This solution was then titrated with a 0.005 M NaOH solution.

The remaining oxidation solution was added to 5 mL ethylene glycol and stirred for 30 min to terminate the periodate oxidation reaction. This mixture was placed in a dialysis bag (3 kDa), dialyzed with deionized water for 48 h, and then concentrated to 10 mL by rotary evaporation. NaBH_4_ (35 mg) was added, and the sample solution was neutralized with 50% acetic acid to obtain a pH of 5.0. Next, the solution was dialyzed with deionized water for 48 h, and the composition was determined after hydrolysis and derivatization of the alcohol product using the aforementioned GC–MS.

#### 3.4.6. NMR Analysis

Twenty milligrams of HPP-1 were dissolved in 1.0 mL of D_2_O and placed in a nuclear magnetic tube. ^1^H-NMR and ^13^C-NMR analyses were conducted using a Bruker AM-600 nuclear magnetic resonance instrument.

### 3.5. Immunomodulatory Activity of HPP-1

#### 3.5.1. Cytotoxicity Test

The MTT assay was used to investigate the effect of HPP-1 components on the survival rate of macrophages [64]. In brief, 5 × 10^3^ cells/mL of macrophages were inoculated in a 96-well cell plate and placed in a 37 °C and 5% CO_2_ incubator for 24 h. After discarding the supernatant, cells were incubated for another 24 h. Then, 100 μL each of 0.5 mg/mL thiazole blue MTT solution and DMSO solution was added, and the absorbance (OD) was measured at 570 nm using a microplate ELISA reader (BioTek, Winooski, VT, USA).

#### 3.5.2. Phagocytic Ability

The phagocytic activity of HPP-1 in RAW264.7 cells was investigated using a neutral red assay [65]. Briefly, 100 μL of 5 × 10^4^ cells/mL macrophages were added to a 96-well cell culture plate. After culturing in a 5% CO_2_ incubator at 37 °C for 24 h, the supernatant was discarded and 100 μL of 0.1% neutral red solution were added to each well. After incubation for 1 h at 37 °C, a new supernatant was discarded, and the cells were washed with PBS. Next, 100 μL of the cell lysate acetic acid-absolute ethanol (1:1, *v*/*v*) was added. After 2 h of incubation, the absorbance at 540 nm was measured using a microplate ELISA reader to estimate the phagocytic rate.

#### 3.5.3. Effect of HPP-1 Components on the Secretion of NO, TNF-α, and IL-6

The Griess method was used to determine the amount of NO produced by the samples [66]. An ELISA reader was used to detect the absorbance of each well at a wavelength of 540 nm. The ELISA kit was used to detect immune factors secreted by RAW264.7 cells according to the manufacturer’s instructions. Absorbance was measured at 450 nm using a microplate reader, and standard curves were used to calculate cytokine concentrations [67].

### 3.6. Immunomodulatory Mechanism of HPP-1

#### 3.6.1. Effect of HPP-1 Components on the Expression of iNOS, TNF-α, and IL-6 mRNA

RT-PCR was used to assess the effects of HPP-1 components on the secretion of macrophage RAW264.7 immune factors at the RNA level [67]. The cells were lysed, and RNA was collected according to the manufacturer RNA total extract kit instructions. Five microliters of the resulting RNA were electrophoresed on a 1% agarose gel. Nanodrops were used to quantify RNA concentration and purity. Next, 3 μg of complete total macrophage RNA was used for reverse transcription into cDNA.

SYBR Green prestaining was used for quantification [66]. The corresponding reagents were added to a PCR 8-tube to form a 20 μL reaction system according to the manufacturer’s instructions. Using GAPDH as the internal reference gene, the 2^−∆∆Ct^ method was used to analyze the expression of the target gene. Gene primer sequences for GAPDH, iNOS, IL-6, and TNF-α were obtained from the NCBI GenBank database (Table 1).

#### 3.6.2. Western Bolt Analysis

Western blot analyses were performed to quantify total protein and phosphorylated protein kinases [68]. A chemiluminescence (ECL) kit was used to perform densitometry of each protein.

#### 3.6.3. Receptor of HPP-1 on RAW264.7 Cells Involving in Immunomodulation

RAW 264.7 cells (1 × 10^5^ cells/mL) were loaded onto a 96-well plate to determine the mechanism by which HPP-1 modulates immunomodulatory function. After 24 h of incubation, the cells were pre-treated for 1 h with 5 g/mL of anti-MR, anti-GR, anti-SR, anti-CR3, anti-TLR4, and anti-TLR2 antibodies before being stimulated with 400 g/mL of HPP-1. The control group was treated with HPP-1 (400 μg/mL) alone, and the positive group was treated with lipopolysaccharide LPS (2 μg/mL). The negative control group consisted of serum-free culture medium. After culturing for 1 h, cell supernatants were collected, and NO and TNF-α levels were quantified as previously described [62] to determine the involved receptor.

### 3.7. Statistical Analysis

All experiments were repeated three times, and the results are presented as mean ± SD. Data were analyzed using IBM SPSS 19.0 program (SPS Inc., Chicago, IL, USA). Differences among groups were assessed using one-way ANOVA. Results were considered significant at a *p*-value < 0.05.

## 4. Conclusions

In this study, a novel pectic polysaccharide (HPP-1) with a molecular weight of 59,024 Da was identified. Mannose, rhamnose, galactose, arabinose, and fucose were the primary monosaccharide components of HPP-1. The presence of 1,4-D-Gal, 1,6-D-Man, 1,3-L-Ara, and 1,2-L-Rha connections was also confirmed. In vitro bioactivity experiments revealed that HPP-1 has significant immunomodulatory activity, which might be mediated via phagocytic promotion and might increase NO, IL-6, and TNF production. The immunomodulatory effect of HPP-1 is primarily mediated through the NF-κB and MAPK signaling pathways via the GR receptor. Therefore, HPP-1 has potential applications in immunological illness treatment and as a functional food.

## Figures and Tables

**Table 1:** Primers for qRT-PCR.

Primers		Sequences (5′——3′)
GAPDH	Forward	AGGTCGGTGTGAACGGATTTG
	Reverse	GGGGTCGTTGATGGCAACA
TNF-α	Forward	CAGGCGGTGCCTATGTCTC
	Reverse	CGATCACCCCGAAGTTCAGTAG
IL-6	Forward	CTGCAAGAGACTTCCATCCAG
	Reverse	AGTGGTATAGACAGGTCTGTTGG
iNOS	Forward	GTTCTCAGCCCAACAATACAAGA
	Reverse	GTGGACGGGTCGATGTCAC

## Data Availability

The data supporting the findings of this study are available from the corresponding author upon reasonable request. Informed consent was obtained from all subjects involved in the study.
